# Institutional Responses to Voluntary Assisted Dying: An Empirical Study in Victoria and Western Australia

**DOI:** 10.1007/s11673-024-10418-z

**Published:** 2025-08-15

**Authors:** C. M. Haining, L. Willmott, B. P. White

**Affiliations:** Australian Centre for Health Law Research, Faculty of Business and Law, Queensland University of Technology, GPO Box 2434, Brisbane, QLD 4001 Australia

**Keywords:** Voluntary assisted dying, Regulation, Institutions, Local, Qualitative, End-of-life

## Abstract

Patients seeking to access voluntary assisted dying (VAD) are necessarily at the end of their lives. Hence, they are likely to be concurrently receiving care from institutions (community nursing services, health services, palliative care services, and aged care facilities) with different levels of participation in VAD. This article reports on the various institutional approaches to VAD based on eighteen semi-structured interviews with regulators from Victoria and Western Australia, representing fifteen institutions with varying levels of support for VAD. We generated five main themes from reflexive thematic analysis of the interview data: settling on a position; operationalizing a position and determining level of involvement; local policy decisions on specific VAD activities; transparency; and navigating pluralism and accommodating diverse views. Overall, our findings revealed significant diversity in how institutions approached various VAD activities and that an institution’s position on VAD (whether it supports it or not) does not necessarily indicate the extent to which it will facilitate access to VAD. These findings may have relevance for institutions seeking to implement (or revise) their approach to VAD and suggest that greater transparency about institutional approaches to VAD is needed to enable people to make informed decisions about where to seek care.

**Supplementary Information:**

The online version contains supplementary material available at 10.1007/s11673-024-10418-z.

## Background

After decades of failed legislative attempts (Willmott and White 2021), all Australian states and the Australian Capital Territory have now passed voluntary assisted dying (VAD) laws, with the Northern Territory likely to follow (Northern Territory Government 2024). Notwithstanding jurisdictional variation, the “Australian model of VAD” is characterized by lengthy and prescriptive legislative frameworks (Waller et al. 2023). Each jurisdiction limits access to adults at the end of their life with decision-making capacity who satisfy strict eligibility criteria. Eligible individuals can access VAD medication dispensed by a centralized statewide pharmacy service via self-administration (the person takes the VAD substance themselves) or practitioner administration (practitioner administers the VAD substance). A centralized navigation (or support) service also exists in each state, which serves as a point of contact for the community, health practitioners, and health services seeking information about, or assistance with, VAD.

Individuals seeking VAD are necessarily at the end of their life and often receive care from healthcare institutions, including community nursing services, aged care facilities, stand-alone palliative care units/hospices, and health services. Australia’s approach to regulating institutional participation in VAD varies across jurisdictions. No legislation requires an institution (or its staff) to participate directly in VAD; however, some jurisdictions impose legislative obligations on institutions to help facilitate access (Waller et al. 2023). In Victoria, Western Australia, and Tasmania, VAD laws are silent on institutional participation, although policy guidance is available (Waller et al. 2023). Conversely, obligations are placed on institutions in the Australian Capital Territory, South Australia, Queensland, and New South Wales regarding the transparency of the institution’s position, providing access to information, permitting VAD personnel to be on-site, or facilitating transfers to other facilities. These obligations vary across jurisdictions and settings (e.g., residential or non-residential).

All jurisdictions still permit healthcare institutions to have discretion regarding their local regulatory approach (i.e., regulation at the institutional level), including the extent to which they directly participate in VAD (if at all). The designated implementation period in each jurisdiction (i.e., the period between the passing of the law and the time it took effect) provided an opportunity for institutions to develop local resources, policies, and processes to assist with service delivery (O’Connor et al. 2021; White et al. 2019). Departments of health developed a suite of resources (guidelines, factsheets, and policy templates) and hosted implementation conferences and/or webinars (Close et al. 2021) to support, *inter alia*, the development of institutional responses. Departmental guidance commonly recommends (and, in some cases, mandates)^1^ that institutions develop local policies and procedures, accounting for the institution’s scope of practice, resourcing, and staff capability. Such guidance also encourages institutions to consider their staff’s willingness to be involved, recognizing that some staff may wish to claim a conscientious objection (i.e., when a person declines to participate in VAD due to their personal beliefs, values, or moral concerns) and limit their participation.

Guidance similarly recognizes that an institution may object to the VAD process (or aspects of it) if it is inconsistent with its values and, as a consequence, limit its participation. Such non-participation has been referred to as having an institutional objection (IO)(White et al. 2021) and has been claimed on both religious and secular grounds (e.g., perceiving VAD to conflict with medical ethics/care or principles of palliative care) (Close, Willmott, et al. 2023; Close, Jeanneret, et al. 2023; White et al. 2023a). Whether there is a normative basis for permitting IO is contentious and heavily debated (e.g., Annas 1987; Durland 2011; Flynn and Wilson 2013; Sepper 2012; Shadd and Shadd 2019; Sumner 2019, 2021). Emerging evidence in Australia suggests that IO can have a profound effect on patients, caregivers, and health practitioners (Haining, Willmott and White 2023; Victorian Voluntary Assisted Dying Review Board 2022, 2023; Western Australia Voluntary Assisted Dying Board 2023; White et al. 2023a, b; Willmott, White and Haining 2025), the impacts of which may be more pronounced than when an individual claims a conscientious objection. One Australian study conducted with family caregivers of people seeking VAD found that IO resulted in “delays, transfers, choices between progressing [a VAD] application and receiving palliative or other care, and adverse emotional and relationship experiences” (White et al. 2023a, 9).

Institutional objection is not a fixed construct. Indeed, the extent of non-participation is highly variable. In some cases, non-participation may be the product of pragmatic considerations (e.g., scope of practice, resourcing, and staff capability) rather than an IO. To date, non-participation by institutions has largely been reported as impacting three main aspects of the VAD process: (1) eligibility assessments within the institution; (2) delivery of the VAD medication to the institution by the statewide pharmacy service; and/or (3) the ability of people to take the VAD medication in the institution or have the VAD medication administered to them in the institution (White et al. 2023a). However, instances have also been described where staff have been prohibited from discussing VAD (where ordinarily permissible), certifying death certificates, and being present at a person’s home (at the person’s request) during self-administration, as well as individuals being refused admission into a facility after communicating an intention to seek VAD (White et al. 2023a). In some cases, the institution as a whole may not object to providing VAD, but individual units within the institution (such as palliative care units) may (Holmes et al. 2022; Waran and William 2020). Institutions not offering VAD in circumstances where the institution is yet to form a position on VAD have also been reported (Adshead 2021).

Government departments have issued high-level guidance to assist institutions in meeting their legislative (and, in some cases, accreditation) requirements and recommend that non-participating institutions have processes in place to deal with VAD requests to avoid obstruction (Close, Willmott, et al. 2023). National and state-level peak bodies have also issued guidance to inform local responses (Close et al. 2021; Close, Willmott, et al. 2023). Such guidance provides advice about developing local responses that are consistent with the peak body’s (and, by virtue of its membership, the member organization’s) overarching values or commitments. In recognition of the diversity of views within its membership, peak bodies’ guidance is often non-prescriptive, enabling tailoring to the institutional context. For example, Catholic Health Australia’s position on VAD is that while their “services strive to ensure that those in [their] care die in comfort and with dignity,” they will not “assist [consumers] to end their own lives or provide euthanasia” (Catholic Health Australia 2019) —a position consistent with their Ethic of Care (Catholic Health Australia 2001). Catholic Health Australia has issued a series of general recommendations and information packages, which can be adapted by members (who are primarily, but not exclusively, Catholic organizations) to assist them in developing local responses that would conform with the Catholic ethos.^2^

Local regulatory responses to VAD in Australia are currently underexplored. While insights into an institution’s regulatory approach can sometimes be gleaned from publicly-facing policies, the level of detail in such policies varies significantly. Policy analysis on publicly facing Victorian policies identified that many institutions’ policies merely detailed the institution’s broad position on VAD, offering limited insight (if any) into the institution’s scope of participation (Close, Willmott, et al. 2023). This lack of transparency limits the ability of patients to make informed decisions about their care and which institutions to attend or reside in (Close, Willmott, et al. 2023).

While some empirical research has explored local regulatory responses to VAD in Australia, existing literature has mainly focused on single institutions and implementation efforts before the relevant VAD laws commenced (e.g., Auret et al. 2022a,b; Digby et al. 2022; Fuscaldo et al. 2021; Sellars et al. 2021), with limited literature focusing on the nuances of institutional responses (Booth et al. 2021) and how these may have evolved. There is also a body of work that reports on the more focused issue of IO (White et al. 2023a, b; Haining, Willmott and White 2023; Willmott, White and Haining 2025). This paper, which reports on findings from a qualitative study into VAD in Western Australia and Victoria, aims to step beyond this existing research by (a) analyzing multiple institutions’ approaches to VAD, (b) reflecting on the breadth of choices about levels of participation in VAD, and (c) shedding light on actual practices after VAD laws have commenced.

## Methods

This study reports on a subset of semi-structured interviews from a broader qualitative study previously reported on elsewhere (White, Haining, and Willmott 2025) with participants from Victoria and Western Australia (where legislation is silent on institutional participation). Participants were considered to be “regulators” if they regulated VAD behaviour by steering or guiding behaviour indirectly or directly (Braithwaite, Coglianese and Levi-Faur 2007). This approach was informed by Julia Black’s broad definition of regulation.^3^ This broad construction of regulator recognizes that state and non-state entities (such as healthcare institutions) can regulate (White, Willmott and Close 2022). This observation is significant for this paper because it reports on a subset of our regulator interviews, namely those involved in local regulation (i.e., at the level of the institution), whether or not they are state or privately-funded entities.^4^ Such participants will hereafter be referred to as institutional representatives.

To recruit institutional representatives, we adopted a purposive approach to sampling to capture perspectives across multiple settings (i.e., health services, aged care, palliative care, and community nursing), with representation from institutions with different positions on VAD (e.g., supportive and non-supportive), funding models (public and private), and locations (metropolitan and regional). To do this, we undertook a scoping exercise to identify potentially relevant institutions. Firstly, Casey Michelle Haining (CMH) generated a list of all relevant institutions in Victoria and Western Australia and tried to identify the institution’s position on VAD, drawing on information in the public domain, including position statements/policies, parliamentary submissions, and annual reports. Previous scoping work carried out in the Victorian context, including an institutional policy analysis (Close, Willmott et al. 2023) and a provider map of aged care facilities (Dying with Dignity Victoria n.d.), was also drawn on. Prospective participants were either directly or indirectly (via their organization) contacted or recruited via professional networks and peak bodies.

Regulator interviews were conducted by the authors with one to three interviewers, with one interviewer taking the lead in each interview. Each interview guide was tailored to reflect the participant’s regulatory role but broadly covered the same domains, namely the nature of the participant’s regulatory role, perceptions of the regulator’s regulatory approach, and VAD regulation more generally. Interviews were audio-recorded and transcribed by a professional transcription company. Field notes were written following each interview to enable reflexivity. CMH was present during each institutional interview, de-identified and sent each transcript back to participants for review. Each participant was requested to provide a copy of any internal policies and resources relating to VAD for data triangulation purposes.

Transcripts were imported into NVivo Plus 12 to store and manage data. Reflexive thematic analysis was used to analyse the entire dataset due to the flexibility of the approach, its ability to explore deep and nuanced interpretations of the data, and avoid reduction (Braun and Clarke 2006; Braun and Clarke 2022). As is common with large datasets, for the purposes of this paper, we reanalysed relevant codes and themes from our original analysis with a focus on local regulation to address the overall research question: What is the nature of VAD local regulation and how do such local responses vary?

Initially, CMH familiarized herself with the data by listening to each interview’s audio and reading each transcript, before coding the data. For the purposes of this paper, CMH reviewed the relevant codes relating to local regulation from the larger data set and generated themes. Interview data were triangulated with local policies and procedures (when provided) and information in the public domain (where possible). CMH’s generated themes were then reviewed and refined by Lindy Willmott and Ben P White**,** with the final themes reported here agreed upon by all authors.

This study was approved by Queensland University of Technology’s Human Research Ethics Committee (Ref: 20000002700).

## Results

Our data from institutional representatives represent a subset of data obtained from a larger set of regulator interviews across two periods—May to September 2022 for Western Australian regulators and September 2022 to July 2023 for Victorian regulators (White, Haining, and Willmott 2025). Overall, we conducted forty-seven regulator interviews with fifty-five individuals via Zoom or telephone, with one to three participants per interview. For this article, we draw on the data relating to local regulatory approaches of fifteen institutions (some with multiple sites) based on data from eighteen interviews with seventeen participants (see table 1). One participant took part in two interviews due to their involvement with two local responses. Interviews with institutional representatives ranged between 57 and 152 minutes. Each institution had representation from at least one participant who was intimately familiar with the institution’s approach to VAD, including having insight into its development, implementation, and/or operation. Each institutional case has been de-identified to protect the confidentiality of the participants and the institutions they work for.^5^ However, to assist with result interpretation, some general characteristics have been provided in table 2. For the purposes of classification, we categorize institutions as having an IO if they elect not to offer the full suite of VAD activities to the extent to which their capability permits. We do not consider limiting VAD to particular individuals (e.g., own patients or catchment) to constitute an IO.

**Table 1:** Participant demographics

Demographic		Institutional representatives (n=17)
**Location**	Victoria	10
	Western Australia	7
**Gender identity**	Female	8
	Male	9
**Age**	–49	4
	50–59	6
	60–69	5
	70+	2

**Table 2:** Characteristics of institutions

Institution type	Catchment	Multiple sites	Funding	Religious-affiliation	Existence of IO
Health service provider	Metro	Y	Public	N	N
Health service provider	Metro	Y	Public	N	N
Health service provider	Metro	Y	Public	N	N
Health service provider	Metro	Y	Public	Y	Y
Health service provider	Metro and regional	Y	Private	Y	N
Health service provider	Regional	Y	Public	N	N
Health service provider	Regional	Y	Public	N	N
Health service provider	Regional	Y	Public	N	N
Aged care	Metro and regional	Y	Private	Y	Y
Aged care	Metro and regional	Y	Private	Y	Y
Aged care	Regional	N	Private	N	N
Community palliative care	Metro and regional	Y	Private	N	Y
Community palliative care	Metro	Y	Private	N	N
Stand-alone palliative care unit	Regional	N	Private	N	N
Stand-alone palliative care unit	Metro	Y	Private	Y	Y

**Fig. 1:**
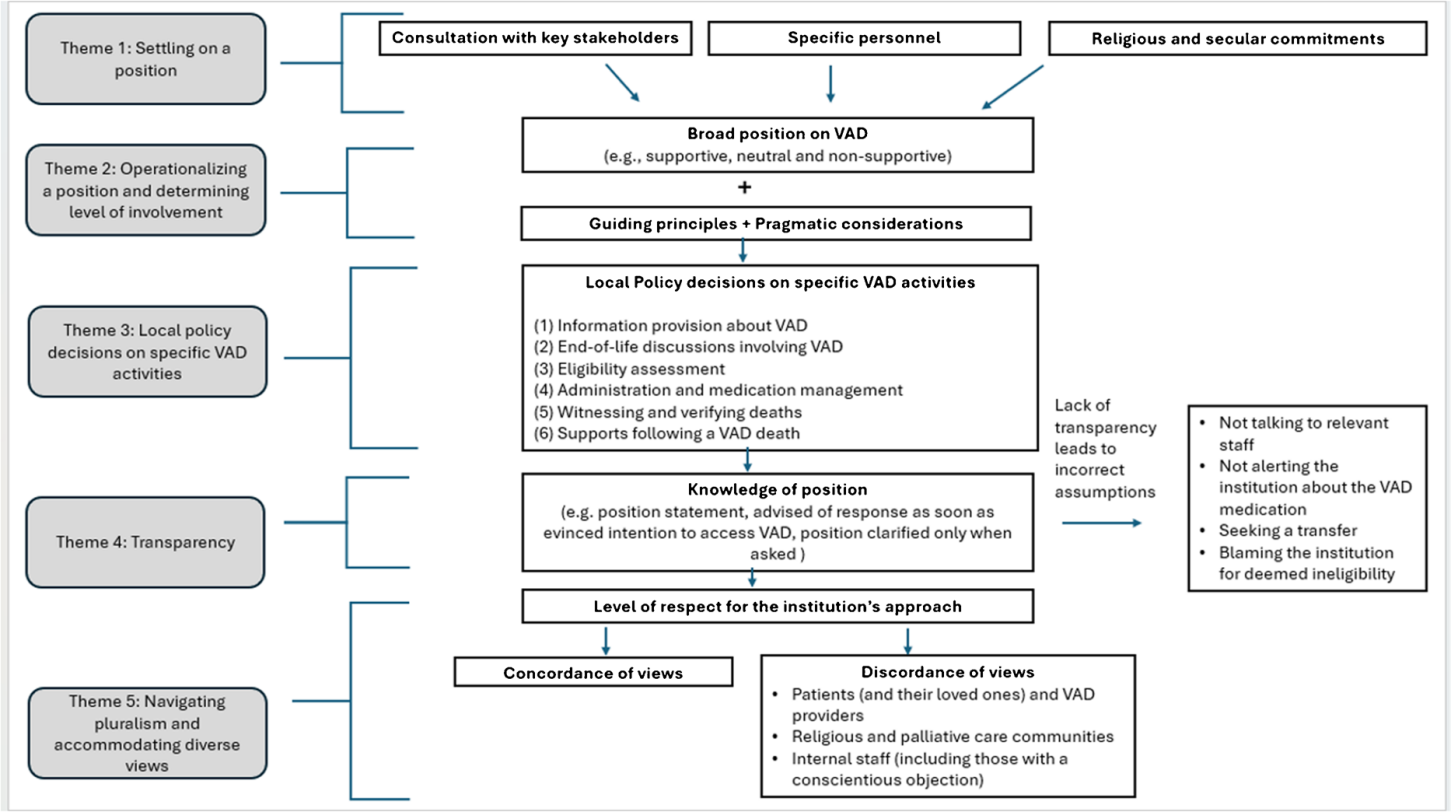
Thematic schema

As a result of data analysis, we generated five main themes—settling on a position; operationalizing a position and determining level of involvement; local policy decisions on specific VAD activities; transparency; and navigating pluralism and accommodating diverse views—that will now be examined. The thematic schema in Fig. 1 shows the relationships between these themes.

### Settling on a Position

Most participants reported that considerable preparatory work informed the institution’s position on VAD, which determined whether it supported VAD in principle. Most institutions were proactive in formulating their position when VAD laws were being considered or during the implementation period after the law passed, reporting that their position derived from extensive consultation with key stakeholders (e.g., executive, church representatives, peak bodies, staff, and consumers). Some institutions used the consultation to develop (or interpret) broader frameworks (e.g., theological frameworks) that were then translated into a position on VAD. In one institution, consultation was not deemed necessary to determine the organization’s position on VAD.

*Interviewer: Board engagement or not on that issue?*

*Respondent: No … [the decision to offer VAD] would be seen here as operational. *[#9] [Non-IO, Private]

Views of certain personnel within the institution were sometimes thought to influence the institution’s position and/or approach to implementation.

*What I didn’t appreciate necessarily is how much an individual leader’s views can translate into an [institution’s] processes … if leadership are agnostic or even ambivalent about the legislation, there is more likelihood that one gets a good enough balance between respecting patient[s’] decisions and autonomy, whilst ensuring a positive and safe institutional culture around a controversial issue*. [#7] [Non-IO, Public]

Each institution acknowledged the lawfulness of VAD and the need to respect a person’s choices. However, some institutions that did not support VAD limited their participation. Opposition to VAD was stated to be on religious and secular grounds (e.g., inconsistent with palliative care philosophies).

*We don’t want to participate in VAD because of the long-held [religious] position and ethic that prefers to journey with a person to the end of life rather than to hasten the death of a person. We would prefer quality palliative care. *[#16] [IO, Private]

Conversely, institutions that supported VAD often framed their support in terms of an overarching commitment to respecting patients’ choices. For some institutions, this stemmed from their long-standing involvement in palliative care.

*Where [Institution X’s] position has come from [is], particularly from the palliative care team … which is around supporting choice and control … [T]his is this client’s illness, this is their trajectory, this is their journey however you wish to describe. And we work with that person in the best way that they would like to be worked with and with the choices that they would like to be able to make. *[#5] [Non-IO, Private]

### Operationalizing a Position and Determining Level of Involvement

In addition to an institution having a broad position on VAD (i.e., supportiveness or not of VAD), institutions needed to operationalize this and make decisions about the extent of their level of support for patients wishing to access VAD. Such an approach appeared to be informed by guiding principles and pragmatic considerations.

As was the case in relation to forming a broad position on VAD and determining whether the institution supported VAD in principle, participants identified several guiding principles that informed their degree of participation in the VAD process. Such principles have been broadly categorized in table 3; however, how these principles were framed varied across institutions. In cases of objecting institutions, values extended to palliative care and religious commitments that needed to be accounted for and balanced with the need to respect a person’s autonomy and the need to avoid obstruction. Different approaches were taken by objecting institutions to reconcile this, including by framing VAD as a “life choice” (and therefore something to respect) and/or ascertaining the level of complicity the institution was comfortable with.

**Table 3:** Guiding principles informing local policy decisions

Guiding principles	Sample quote(s)
Person-centred care and respect for patient autonomy	*We absolutely hold true to the fact that we have this person at [the] centre... it’s not really about us; we know we work under limitations, but we need to work with our patient in whatever [way] to reduce the barriers*. [#8] [IO, Public] *What was very advantageous and … has certainly served us really well from the outset was to see voluntary assisted dying as being a life choice... it was always anticipated that we would encounter people who were on the VAD journey at some point or another... So, essentially, it was always a very strong commitment on the part of the organization to in no way frustrate or be perceived to be interrupting, making things difficult for people.* [#15][IO, Private]
Staff wellbeing and safety	*One of the guiding values was the well-being of staff, with [Institution X] wanting to ensure that all staff, regardless of their views or participation, felt safe, well-supported, and respected. This guided the more detailed parts of process implementation.* [#7][Non-IO, Public]
Religious (or palliative care) ethos and complicity	*The concept of participating is something that we tried to give a bit of definition to … we would not participate in the sense that we would not facilitate [VAD], so that we become complicit in the whole process. So, we wouldn’t get in the way of anybody. But we wouldn’t go out of our way to make it easy for this kind of thing to happen.* [#16][IO, Private]

Pragmatic considerations, including the nature and size of the service, the institutional type (e.g., health service or aged care setting), the nature and capacity of the institution’s staff, reputational risk, and cost, also informed the nature of an institution’s approach (see table 4). Such considerations also influenced the limitations (if any) that the institution put on its delivery of VAD services. Such limitations included whether institutions would limit provision to business hours, limit access to individuals within the institution’s region, limit access to individuals who had an affiliation with the institution or its staff, or would only allow external personnel to participate in VAD provision (as opposed to its own staff).

**Table 4:** Pragmatic Considerations Influencing Degree of Participation

Pragmatic considerations	Sample quote(s)
Nature and size of the institution	*Not participating was probably not necessarily a pragmatic option for a public health system.* [#7] [Non-IO, Public] *When you’ve got such a big service and [you occupy a portion of the] private market, if you are not involved in voluntary assisted dying, you’re going to need to have a referral process in place because there’s no way you’re not going to have people enquiring about this*. [#6] [Non-IO, Private]
Setting of the institution	*Our residents in our residential facilities live in their home. We work in their homes; they don’t live in our workplace. So that’s a fairly careful distinction we try to make.* [#16][IO, Private]
Nature and capacity of the staff	*Medical staff that we do have here as part of our service are not involved in that process; they are registrar level. So, I think that would preclude them at this point in time from being involved. And so yeah … it’s support in whichever way that looks like in the client’s situation.* [#5] [Non-IO, Private] *So [Institution X] [could not see] everybody at all times, because [Institution X] really couldn’t say that [they] would have the medical staff to provide that level of service. So [Institution X adopted] … a case-by-case situation. I mean, [Institution X] only has to have one doctor on extended leave or sabbatical or whatever, and that [could impact] on the programme*. [#10] [Non-IO, Public]
Reputational concerns	*One of the things that our board were very clear about was that we were not to have what they called VAD tourism. So, we needed to ensure that anyone who is accessing VAD actually is from [our] region*. [#11] [Non-IO, Private] *Reputationally, it is awkward that—and I think it’s a bit like—we had nothing to do with you, and now we’re going to have a relationship with you in this scenario*. [#6] [Non-IO, Private]
Financial disincentive	*So, whilst it could be argued [that] you’re being restrictive, [Institution X is] being [restrictive] to our own patients... I don’t see this change happening in the next couple of years. There’s no money in voluntary assisted dying, so it’s not like you can make a profit centre.* [#6] [Non-IO, Private]

### Local Policy Decisions on Specific VAD Activities

In addition to decisions about the broad level of involvement (e.g. provision of VAD for all patients or only the institution’s patients), institutions also needed to make policy decisions about different aspects of the VAD process (see Supplementary Material 1), which were similarly informed by the institution’s position and the aforementioned guiding principles and pragmatic considerations.

#### 1. Information Provision about VAD

While only a subset of institutions provided general information about VAD (or made it readily available), all institutions were prepared to provide at least some information about VAD upon request, albeit to varying extents. For many of these institutions, this included (and was sometimes limited to) details of the statewide care navigator service (typically in the form of a handout), which could be contacted for further information. Some institutions would facilitate contact with the care navigators on behalf of the person in all (or particular) circumstances. In most institutions (including some objecting institutions), the care navigators were permitted on-site and treated like private visitors. For institutions with a dedicated VAD liaison (i.e., an individual who was the point of contact for VAD in the organization), information about VAD was provided via that liaison (rather than the care navigators, albeit not exclusively). Sometimes, the VAD liaison was a designated VAD coordinator; in other cases, it was an organization’s manager or a designated clinical lead (e.g., a palliative care physician).

#### 2. End-of-Life Discussions Involving VAD

Many institutions acknowledged the inevitability of people raising VAD with their staff and the need for internal processes to support such conversations. Staff were made aware of legal restrictions with respect to raising VAD.^6^ Many participants observed that when a person raised VAD, it was a good opportunity to discuss the person’s values and preferences more broadly and explore their desire to hasten death. Such conversations were not unusual for many institutions’ staff, who were often considered well-placed to engage in such discussions.

For objecting institutions, while staff were generally permitted to have such conversations with patients, local policies typically required staff to be clear about the limits of their involvement in VAD. In doing so, many participants emphasized the need to respect a person’s choices and reinforced that they would not abandon someone seeking VAD. In some institutions (whether or not they had an IO), an escalation approach existed whereby people requesting VAD would be connected with dedicated VAD liaisons. Other institutions referred patients to the care navigators.

#### 3. Eligibility Assessments

Institutions’ approaches to eligibility assessments varied. Institutions supporting VAD would assist patients in accessing VAD providers—for example, by facilitating contact with willing providers within the institution (or visiting specialists). Where the institution did not have medical staff (or VAD providers), care navigators would assist the patient in accessing a VAD provider, or patients would access willing providers on their own accord (e.g., via their general practitioner).

For objecting institutions, staff were not permitted to conduct VAD eligibility assessments in the course of their employment with the institution, even if qualified to do so. However, in such institutions, staff could provide clinical and prognostication information to other practitioners (including VAD providers) to help inform the VAD eligibility assessment. In the objecting aged care settings examined, institutions would permit external assessors on site to make assessments, but this was not allowed in other objecting settings.

#### 4. Administration and Medication Management

Institutions varied regarding how much they would permit self or practitioner administration on their premises.

- Self-administration

In all but one of the institutions examined that had sites (i.e., not community nursing), the VAD medication could be brought on-site, and self-administration could occur on the premises. Most institutions also indicated that the statewide pharmacy service would be permitted to dispense the VAD medication on-site. Such permission was justified on the basis that the VAD medication was ultimately the person’s property and, therefore, should not be unnecessarily interfered with. Many institutions had strict protocols in place in relation to the VAD medication and, in some cases, the ability of their staff to handle the substance, which was prohibited in some institutions.

When self-administration took place in a health service or inside a palliative care unit, many participants observed that restrictions and controls were in place. These were often justified on safety grounds. Some participants reported that their institution encouraged patients to take the medication at home (where possible), with some institutions having processes in place to assist with discharge into the community, including offering to put the person in contact with community nursing services.

However, participants conceded that discharge was not always possible (or desirable), and consequently, the person would be permitted to take the medication on-site. Institutions varied with respect to their VAD medication storage requirements. Some institutions allowed patients to have their medication in a locked drawer close to their bed, whereas others required the medication to be locked elsewhere. Moreover, some institutions required patients to sign documentation and advise precisely when they would take the medication. In some cases, administration had to occur during set times or in the presence of specific staff members (who, in some institutions, were permitted to prepare the VAD substance). In other settings, stringency around time and personnel present did not exist. However, institutions encouraged people to notify them about their anticipated time of death so that they could assist with administrative and/or clinical arrangements.

(b) Practitioner-administration

Practitioner administration was not permitted at every institution. In all participating aged care facilities, practitioner administration was allowed on-site. This practice was also permitted at non-objecting health services and a stand-alone palliative care unit. Institutions that did not have VAD providers on staff (or did not have enough) needed to credential visiting doctors to facilitate administration, which was one reason (albeit not always exclusively) why practitioner administration was limited at certain institutions. In health services with VAD providers, practitioner administration was generally restricted to those staff members to the extent capacity permitted. At one institution, it was possible to credential a patient’s general practitioner to administer VAD to a patient if there was a long-standing relationship, despite having a pool of VAD providers available.

(c) Presence during administration

Some institutions required specific staff to be present during administration as a form of support. Participants also acknowledged that individuals may request that certain health professionals and/or carers be present during administration, and institutions varied in the extent to which this was permitted. In some cases, presence during administration was left to the discretion of individual staff members. In other cases, the institution would allow its staff to be present on a case-by-case basis.

#### 5. Witnessing and Verifying Deaths

Institutions varied regarding the extent to which they would permit staff to witness and verify VAD deaths. Witnessing is required at multiple stages of the VAD process, including at the final request stage and/or during practitioner administration. In some institutions, staff were not permitted to witness. In other institutions, staff were allowed to witness and/or facilitate contact with an advocacy service that could provide volunteer witnesses. Some institutions also indicated they would verify deaths; however, it was unclear whether all institutions permitted this.

#### 6. Supports Following a VAD Death

Support for families following a VAD death was generally available from institutions regardless of whether they supported VAD. In some institutions, this would be the extension of bereavement support offered following any death. In other institutions, dedicated VAD bereavement procedures were in place. Support was also made available to staff following a VAD death, regardless of whether the staff were directly involved. One participant described a situation where pastoral care was extended to a VAD provider who visited the institution for administration.

### Transparency

Institutions varied regarding the extent to which details of their local response on VAD were publicly available. However, no institution had its local policies (where they existed) or detailed information about the extent to which they supported VAD (i.e., activities they took part in or permitted) readily available to the public. Some institutions published VAD position statements (or equivalent), although this was not uniform. Published position statements were often very high-level, articulating, in general terms, the institution’s supportiveness (or not) of VAD. Some participants attributed the failure to publish a position statement to oversight. However, in one institution, there was a deliberate decision not to do so to avoid drawing attention to VAD.

*Interviewer: … [So, your approach is] not necessarily to advertise the fact that you won’t be providing VAD … [but] just clarify [your scope of participation] when [asked], rather than to signpost it.*

*Respondent: Yes, absolutely.* [#15] [IO, Private]

Regardless of the approach, participants reported that if asked about the institution’s position, they would detail the extent of their participation to permit people to make informed decisions about attending (or remaining in) the institution. In some institutions, as soon as the patient evinced an intention to access VAD, they would be advised of the institution’s response.

*[We provide] clarity for the customer on [Institution X’s] approach. So, they may have read the [position statement], but that’s words on a page, and they can interpret that how they choose to do so … the approach is that [the leader at the site] meets with the customer and/or their family if that’s what they want, and we have [a] clarifying conversation*. [#12] [IO, Private]

The lack of transparency by institutions in some cases led to individuals seeking VAD and/or their loved ones making incorrect assumptions about the institution’s approach. In the case of objecting institutions, the starting assumption was often that the institution would attempt to obstruct access if it did not support VAD in principle. This manifested in several behaviours including not talking to relevant staff members out of fear they would coerce them out of VAD, not alerting the institution that the VAD medication was in their possession, requesting transfers to other facilities because of fear they would be blocked, and/or a person’s loved ones blaming the institution for their loved one’s ineligibility (table 5).

**Table 5:** Behaviours driven by assumptions about institutions’ positions

Behaviours driven by assumptions	Sample quote(s)
Not talking to relevant staff	*We had one situation where the customer wanted to connect with the chaplain, but the family didn’t want the chaplain to connect with the resident because their perception was that the chaplain would talk the resident out of voluntary assisted dying*. [#13] [IO, Private]
Not alerting the institution about VAD medication	*We’ve got several patients … who don’t even talk about [VAD], but we know they’ve got [the VAD substance].* [#8] [IO, Public]
Seeking a transfer	*We’ve had people in our palliative care unit who want to use it, want to get on the process, but have wanted to go back to their parent hospital before they use it. So, we say to them, “You can stay here if you want to; we’re not going to stop you.”* [#8] [IO, Public]
Blaming the institution for deemed ineligibility	*So, essentially all we had done was just flag that there could be a potential [capacity] issue … So, the coordinating practitioner got involved … [they] reassessed, made a determination that, in fact, there had been a change of capacity, and the person no longer had capacity. So, the substance then needed to be removed... the ramifications of that at that time were really significant because we had a really angry family on our hands who made [a] complaint … And it was a terrible situation because it was terrible for the family, because they perceived that somebody who had wanted it, had been deprived of it*. [#15] [IO, Private] *We’ve found the navigators give an independent voice—because often, you will find people that aren’t going to be eligible. And it’s not us saying, “You’re not eligible,” because you’re in [a religious institution]; they’re ineligible because they’re maybe not a resident, or timing, or all sorts of things.* [#8] [IO, Public]

### Navigating Pluralism and Accommodating Diverse Views

Some participants indicated that just as institutions should respect a person’s right to VAD, the values of an institution, its position and its approach to VAD should be respected by others. However, it was reported that this was not always the case. One participant described a situation where a VAD provider made comments that devalued the institution’s position, even though the institution permitted VAD to take place.

*[There was a case where there was an] inability to understand [our] position … [with the] doctor coming in and saying something like, “You’ve given this person the great gift of dying.” And we go, “No, that’s not what we would have wanted, if we had the choice, we would have preferred palliative care to the end.” But it was—you need to celebrate with champagne … that just left a rotten taste on the part of the staff... I can accept that a doctor needs to do his or her thing, but can we just have … a more sensitive approach to this? … [I]t might just be individuals involved … another doctor coming along might handle that differently*. [#16] [IO, Private]

Some participants noted that some hostility (e.g., from patients, their loved ones, and VAD providers) was averted by working closely with the care navigators, who were aware of the institution’s limits and helped minimize any disruption to the person’s care.

*We’re very clear to the navigators where our lines in the sand are.* [#8] [IO, Public]

However, working relationships with care navigators were not uniformly reported. The absence of such a relationship was considered disadvantageous.

*In my conversations with my counterparts and other service providers, we understand that some of them have … a working relationship with the navigators. That means that if someone has requested a doctor to come in, the navigators will liaise with the facility. We haven’t got that in place at the moment. I have requested it … at least it would give us the heads up.* [#16] [IO, Private]

In a different vein, some participants representing institutions with an IO indicated that the way in which their institution was still able to support VAD (even if not involved in direct provision) was not uniformly accepted. On the one hand, participants reported that some people found it difficult to reconcile with the permissibility of the institution’s position. This was particularly the case among the broader religious community.

*I brought two new chaplains in at one stage, and we were having another VAD discussion and the look on their faces it was like, “Oh yeah, you guys haven’t been through this journey, let me take you through it quickly.”* [#13] [IO, Private]

Despite potential backlash, however, participants noted that the ability of institutions with an IO to still support patients seeking VAD (to some extent) was preferable to some more restrictive approaches taken by some other institutions with an IO, which participants described as obstructive and impractical.

*It’s very enculturated at [Institution X] that you hold the patient at the centre. And that’s different to some of the other [institutions] who’ve struggled a bit and got themselves into a bit of argy-bargy.* [#8] [IO, Public]

Diversity of views was also evident among staff members of the institution. Indeed, participants indicated that, notwithstanding the extent of involvement of their institution in VAD, some staff may not fully accept the institution’s position (due to its permissiveness or restrictiveness). In cases where an institution did not support VAD, some participants reported that their staff could still participate in VAD outside of their employment with that institution.

*[Staff] may work in another private hospital, or they might work in another public hospital, then there’s a freedom to choose within that whether they participate or not. But … when they work under [Institution X’s] flag … that’s where the non-assessment, etcetera [applies].* [#8] [IO, Public]

Participants also noted that staff were permitted to seek employment elsewhere (and, in some cases, may be managed so they do so).

*We’ve had one or two staff at each of the sites where we’ve had [someone] go through the process who have a strong faith themselves and found it particularly confronting … Broadly speaking, we don’t have extremes within our organization. I think people would likely have either been managed out of the organization in a wider context or would’ve self-selected that, “This probably isn’t a place I want to work,” if they’re of that particular mindset*. [#12] [IO, Private]

In some cases, staff working in an institution that provided VAD would claim a conscientious objection. All institutions supported conscientious objectors and aimed to support them by various means, including adjusting rosters, providing debriefing opportunities, and making support services known and available either within the institution or externally.

*We do have [staff] that have registered concerns … once they understand that there are some legal things that we’ve got to respect, and then our position and how we’ve tried to navigate that, that’s been a little better to deal with … there’s also the conscientious objection clause within the legislation, which we’ve made clear to the people in the training, and we’ve told them how that can be accessed. And that does not impact their job or their position … or anything like that.* [#16] [IO, Public]

In most cases (but not exclusively), individual conscientious objectors were perceived not to frustrate the process and were viewed as sympathetic.

*I’d like to change the word “conscientious objector” in the next legislation because I think it’s quite a negative word. When I say people are conscientious objectors, I’m cringing inside because they’re still sympathetic to the whole process.* [#1] [Non- IO, Public]

While most participants did not report issues with conscientious objectors, multiple participants noted tensions arose when some sites and/or units did not adopt the institution-wide response. Some of these tensions resolved over time, while others did not.

*[There were] implementation issues with a couple of sites that actually said, “Yeah, great system, wonderful thing, but we won’t be doing it here.”* [#6] [Non-IO, Private]

One participant spoke of a particular unit within an otherwise non-objecting institution that refused to offer (and sometimes discouraged) VAD. In some cases, attempts were made to orchestrate transfers out of the unit when VAD was sought, which was considered distressing. Reflecting on a particular experience, the participant recalled:

*[The objecting unit was proposing to move the] patient … [but they] did not have a permit [to access VAD]. [It was] felt that the permit would come through the next day. [The patient] felt [they were] being moved because [they] had to die. So [they were] extremely distressed by that. [The VAD coordinator] got a call from [the patient’s loved ones] [and then] rang the ward to say, “Why are you moving [X]? [They do not] even have a permit.” And the nurse in charge said, “Well, I’ve been told by [one of the] physician[s] that [X] has to move” … that was just so upsetting.* [#18] [Non-IO, Public]

In other institutions, VAD was generally available on all wards/campuses (to the extent there were available doctors). Transfers only occurred due to ward capacity concerns and/or if patients wanted to move to a nicer ward.

*In [Institution X] itself, you can access it wherever you are.* [#1] [Non-IO, Public]

## Discussion

This paper provides insight into local responses to VAD in two Australian jurisdictions across various settings. It supplements current Australian VAD literature that explores the development of local responses (Auret et al. 2022a,b; Booth et al. 2021; Digby et al. 2022; Fuscaldo et al. 2021; Peisah et al. 2023; Sellars et al. 2021), institutional policy analysis (Close, Willmott, et al. 2023), and articles reporting on the impacts of IO (White et al. 2023a, b; Haining, Willmott, and White 2023; Willmott, White, and Haining 2025). This paper supports the conception of holistic regulation (White et al. 2022). It recognizes that regulation is not necessarily limited to distant authorities regulating in a top-down manner. Indeed, non-state entities such as healthcare institutions, regardless of whether publicly or privately funded, are capable of steering or guiding behaviour (i.e., regulating) (Black 2002; White et al. 2022). While these healthcare institutions do not necessarily perceive themselves as regulators, they have been able to exert regulatory force and do have regulatory influence (Oikonomou et al. 2019; White, Haining, and Willmott 2025). Such influence is evident in the significant diversity we observed in relation to local VAD approaches. This diversity is consistent with that observed internationally. Indeed, recent qualitative studies in Canada (Close, Jeanneret, et al. 2023) and New Zealand (Snelling et al. 2023) have reported wide variation in organizational responses to assisted dying. Understandably, context (institutional type, funding model, and denomination) shapes approaches, and variability can be expected. However, our findings suggest that variability can also exist within similar contexts (e.g., variation across institutions with a commitment to palliative care and/or institutions belonging to the same denomination). This points to the influence of local regulation, particularly in contexts such as Victoria and Western Australia, where legislation on institutional participation is silent, permitting greater discretion over local responses.

Moreover, consistent with what has been observed previously (Close, Jeanneret, et al. 2023), specific personnel and institutional culture were found to influence institutions’ approaches to VAD; many of our findings support this. Firstly, most institutional responses reported were informed by consultation (which naturally needs to respond to various stakeholders’ views) and, in some cases, were primarily shaped by certain personnel (typically at the executive level). Similarly, participants reported that peak body guidance was implemented differently among member organizations, implying that local personnel and institutional culture were influential. Finally, the fact that there was evidence of VAD responses being implemented differently across different sites and units within an institution (as opposed to a unified approach) suggests that specific personnel and culture within particular sites/units were likely influential.

Furthermore, our results support previous findings that institutions typically lack transparency in relation to their position on VAD (White et al. 2023a; Close, Willmott, et al. 2023; Close, Jeanneret, et al. 2023; Willmott, White, and Haining 2025). Indeed, not all institutions examined had publicly facing positions on VAD. Where position statements (or equivalent) did exist, they were often framed with a high level of generality and offered limited insight into the practical details of the institution’s response, making it difficult for people to make informed decisions about their care (Close, Willmott, et al. 2023). While some institutions clarified the boundaries of their VAD involvement as a matter of procedure, this was often not done until the person was already within the institution’s care, instead of at the outset when a person was considering care at the institution. In other cases, institutions only provided the desired level of detail on request. Such an approach is undesirable, considering the power and information asymmetry between patients and health practitioners (Close, Willmott, et al. 2023).

In the absence of clarity about the institution’s position, participants reported that some patients made assumptions about the institution’s supportiveness of VAD, which were sometimes inaccurate. Indeed, evidence from the reproductive context suggests that not all patients anticipate that institutions will restrict access to lawful healthcare or appreciate the breadth of restrictions (Stulberg et al. 2019). In a different vein, there is also evidence to suggest that people may assume that VAD is off-limits in an institution because of its religious affiliation, which in some cases has resulted in surreptitious efforts to access VAD (Knox and Wagg 2023; White et al. 2023a).

It is worth noting that to assist individuals in making informed decisions about institutions, some advocacy organizations have tried to map the degree of VAD participation of various institutions and make this information publicly available (Dying with Dignity Victoria n.d.). While these efforts are welcomed, such assistance is limited in scope. Indeed, in the Australian context, to the authors’ knowledge, this mapping currently only extends to select Victorian aged care facilities. Such mapping is also limited to broad positions on VAD and does not capture some of the nuances of institutional responses reported here. Consequently, greater transparency by institutions themselves to proactively communicate their position and processes is needed.

Another significant finding from this study was the degree to which institutions with an IO are actually obstructive or truly objecting. There is no scope in this paper to defend (or contest) the use of the term IO, recognizing that this is subject to ongoing debate (e.g., Annas 1987; Flynn and Wilson 2013; Shadd and Shadd 2019; Sumner 2019, 2021). However, our data suggests that the term IO may be reductionist and, as has been observed in the conscientious objection context, the degree of support (and participation) in VAD is perhaps best conceptualized as existing along a continuum of participation (Haining and Keogh 2021; Rutherford, Willmott, and White 2023). Importantly, our findings suggest that even institutions that “object” to participating in VAD may support access to VAD to some extent, even if operating under constraints. Such a finding is consistent with what has been observed in other contexts (such as abortion) where institutions perceived as restrictive by the public (e.g., due to religious affiliation) may actually support provision of healthcare they object to and, in some cases, more so than institutions who may be perceived as more likely to support provision (e.g., secular public hospitals) (Haining, Bowman-Smart et al. 2023). Although these findings need to be interpreted in the context of our sample (which featured less robustly objecting institutions: see Limitations), our results did suggest that some non-objecting institutions had more restrictive VAD practices (e.g., medication storage requirements, only permitting VAD services during business hours, restrictions placed on the presence of staff during administration, and transfer requirements) than some objecting institutions. Although some of these local policy decisions were influenced by pragmatic considerations (e.g., staff capacity and safety), other practices (e.g., attempted transfer out of a unit) reflected a lack of support within a unit (Rutherford, Willmott, and White 2023).

This finding further reinforces the desirability of institutions to be transparent about their position on VAD and the benefits associated with having details about their local practices publicly available. This would facilitate people making informed decisions and prevent instances where people may avoid institutions based on misplaced assumptions. In the absence of such transparency, people are subjected to what has been described as an “institutional lottery” whereby the nature of the care they receive varies depending on which institution they end up in, the effects of which are compounded by the fact that they cannot anticipate the nature of the care they will receive before being in the institution’s care (Haining, Bowman-Smart et al. 2023). The importance of enabling individuals to make informed decisions is reflected in more recent Australian VAD laws, which impose information provision obligations and distinguish institutional obligations based on setting (imposing greater obligations on residential institutions compared to non-residential) (Waller et al. 2023). However, given the infancy of these provisions, the extent to which such legislative framings influence institutional practices remains unexplored empirically.

A related observation deriving from our findings is that in a pluralistic society, objecting institutions (whether secular or religious) can still operate in contexts where VAD is lawful and can continue to provide treatment or care. We acknowledge that IO behaviours can cause significant distress and frustration for patients, families, and health professionals (Brown et al. 2020; Close, Jeanneret, et al. 2023; Haining, Willmott, and White 2023; Holmes et al. 2018; Snelling et al. 2023; White et al. 2023a, b; Willmott, White and Haining 2025) and our sample did not include institutions with particularly restrictive approaches to VAD which have been identified in the Australian context (Adshead 2021; Cunningham 2021; Victorian Voluntary Assisted Dying Review Board 2022, 2023; Western Australian Voluntary Assisted Dying Board 2023; White et al. 2023a; Willmott, White and Haining 2025). However, our findings suggest that some objecting institutions can reconcile, at times, conflicting values and guiding principles to deliver a model of care that respects patients’ choices (albeit to varying extents) and minimizes barriers to access.

In aiming to strike this balance, institutions were guided by principles such as commitment to delivering person-centred care and more pragmatic considerations such as respecting a patient’s right to information to make informed decisions and continuity of tenure (in cases of aged care facilities). Institutions were supported in this through extensive consultation and reflection on different aspects of the VAD process, which reinforces the significance and benefits of early preparation and an implementation period (Auret et al 2022a; Close et al. 2021).

Participants also described having a working relationship with the care navigators as critical to the delivery of their local response. The benefits of such a relationship were twofold. Firstly, the relationship was perceived to serve as a communication mechanism, so the institution was aware of a person’s intention to undertake VAD and could be prepared. Secondly, it serves as a mechanism where institutions could facilitate contact (whether simply by providing the care navigator service’s contact details or contacting the care navigators on behalf of a person) to an impartial information source and/or an entry point into the VAD system (White et al. 2023c). Having effective centralized coordination systems in place helps to maximize access without directly involving individuals in a causal chain that implicates them in the VAD process, representing a minimally coercive approach to regulating institutions (Carpenter and Vivas 2020).

### Limitations

This study has several limitations. Firstly, while efforts were made to sample across a range of settings purposively, the authors are aware that more restrictive approaches to VAD local regulation exist. Our sample did not capture some of the more restrictive responses, despite attempts to do so. Accordingly, these findings may not reflect the full spectrum of positions on VAD. Secondly, given the small sample size and the influence of context and culture, the transferability of our findings may be limited, particularly in jurisdictions that adopt a different regulatory approach. Thirdly, we primarily relied on interview data to understand each institution’s response. While we requested local policies to assist in triangulating our data, not all institutions had (or chose to provide) these.

Furthermore, the level of detail provided by participants (or contained within institutional policies) about local responses was variable, meaning direct comparisons across institutions could not always be made. Moreover, given that participants were intimately familiar with their institutional response (and, in some cases, were directly involved in its development), this may have influenced how they perceived their organizational response affected patients’, family members’, and colleagues’ experiences. Finally, while we present different institutional “cases” here, our research study was not designed as a case study; instead, we have reported here on a subset of interviews from a larger study. Further research that adopts a case study methodology to explore local responses to VAD is warranted to gain a deeper understanding of institutional responses.

## Conclusion

With the advent of VAD laws, institutions need to determine their position on VAD, and develop and implement local processes to manage VAD requests regardless of their level of support. Our findings suggest that how institutions have operationalized their local response to VAD is highly variable, and great diversity exists across different institutions even within similar contexts (e.g., the same institutional type or institutional values). Significantly, our findings shed light on the ability of some institutions that do not support VAD in principle to deliver healthcare in a manner consistent with their values and still support patient access to VAD (or at least minimize obstruction). However, the lack of transparency and publicly available details of institutional approaches means that these nuances are rarely appreciated, and the ability of people to make informed choices about which institutions to receive care from is limited. Further research and consideration of local responses are required to formulate recommendations about improving VAD service delivery and ensuring impediments to patient access are minimized.

## Supplementary Information

Below is the link to the electronic supplementary material.

Supplementary file1 (DOCX 24 KB)

## Data Availability

Supporting data in the form of quotes has been provided in the manuscript and the supplementary material.

## Footnotes

1 For example, in Western Australia, “WA Health System Entities” are required to have a VAD policy. See (Government of Western Australia: Department of Health 2021).

2 Catholic Health Australia comprises Australia’s largest non-government grouping of hospitals, aged and community care services (approximately 10% of hospitals and aged care in Australia). It has recently extended its membership to non-Catholic organizations (e.g., Uniting Care Queensland): see Wood 2022. Catholic Health Australia has issued a position statement, policy, consumer information, and guidance for visiting medical officers working with Catholic Health Facilities and GPs working in Catholic facilities and recommended a tiered approach to addressing VAD requests. The tiered approach recognizes that different types of staff will have different level of competencies and will have a varying level of exposure to VAD requests. Accordingly, staff in lower tiers may be required to escalate cases to higher tier colleagues in particular circumstances. See Catholic Health Australia 2019, 9.

3 Black defines regulation as “the sustained and focused attempt to alter the behaviour of others according to defined standards or purposes with the intention of producing a broadly identified outcome or outcomes, which may involve mechanisms of standard-setting, information-gathering and behaviour modification” (2002, 26).

4 This is not to suggest that other regulators (e.g., state health departments or national peak bodies) do not have influence at the local level.

5 Please note when reference is made to institutions within quotes (i.e., Institution X), these reflect generic labels rather than a specific case. Hence, reference made to Institution X throughout the dataset is not necessarily referring to the same institution. Moreover, to further protect confidentiality, the number we assigned participants in this paper does not reflect the order provided in table 2 (i.e., the first case referred to table 2 is not necessarily linked with participant #1).

6 All health practitioners are prohibited from raising VAD with patients in Victoria: see *Voluntary Assisted Dying Act 2017* (Vic) s 8. In Western Australia, only medical and nurse practitioners can raise VAD, provided they inform the person about the treatment options and palliative care and treatment options available to the patient and the likely outcomes of that care and treatment at the same time: see *Voluntary Assisted Dying Act 2019* (WA) s 10.
