# Supplementary material for: Institutional Responses to Voluntary Assisted Dying: An Empirical Study in Victoria and Western Australia
Source: J Bioeth Inq. 2025 Aug 15;22(4):863–80. doi: 10.1007/s11673-024-10418-z (PMC12783208; doi:10.1007/s11673-024-10418-z)
Supplement: Supplementary file 1 — Supplementary file1 (DOCX 24 KB) [file 11673_2024_10418_MOESM1_ESM.docx]

**Supplementary Material**

**Table 1: Institutions’ Approaches to Aspects of the VAD Process**

| **Aspect of the VAD process** | | | **Sample participant quote(s)** |
| --- | --- | --- | --- |
| 1. **Information provision** | | Making general information about VAD available | *[When] voluntary assisted dying [was adopted], I introduced it to our residents here … as a change in legislation, not to say, “Do you want to do it?” because I know I’m not allowed to do that … but just so they knew what was going on, like I do [with] every other bit of legislation that changes which affects them … So, I had it as an agenda item so that they were prepared and I had the documentation for them to take away and read.* [#9] [Non-IO, Private]    *We have been deliberate in terms of we haven’t had a proactive approach to promoting it.  We didn’t want to do that … I would say, broadly speaking ... it’s known and understood in the community here ... that it is now available, but it would be down to the individual or to the individual and family to be exploring that*. [#12] [IO, Private] |
|  |  | Providing Statewide Care Navigator Service’s details | *[There is] a little handout [that was produced and is] given to patients explaining why as a [religious] institution [the institution does not] agree, “But here at the bottom is the number of the Care Navigators, but we encourage you to speak to your own doctor”.* [#3] [IO, Private] |
|  |  | Facilitating contact with Statewide Care Navigator | *If someone wanted to access the information, we would provide them with the information. If they wanted a member of our staff to phone the Navigator Service on their behalf, we would refuse to do that because that would be participating. They can do that in their own time with their own resources.* [#16] [IO, Private]  *We leave it to the family and the patient, but look, if English isn’t their first language, they can’t navigate mobile devices, so there’s some disability or problem that’s stopping them from making those connections, we can, in fact, kind of work with the Navigators to give them an opportunity to then contact the patient, or the patient contact them. So that’s a very practical, on-the-ground way of looking at the process.* [#8] [IO, Public]    *There is no problem for us if the person is not able to contact the Navigator Service. We can facilitate that; nobody is again saying, “Uh … you can’t actually contact them on somebody’s behalf,” or do any of those things. The whole idea for us is we will help people to access the appropriate information that they need, and we’ll do that in whatever the circumstances require.* [#15] [IO, Private] |
|  |  | Permitting Care Navigators on-site | *We let the Navigators come in because we feel like it’s a private meeting, a private individual; they’re not there to do a clinical assessment per se … they’re not a trained assessor, they’re not coming with the assessor badge on.* [#8] [IO, Public] |
|  |  | Information about VAD by a dedicated liaison within the institution | *To keep the continuity up because the way my role is structured … I have a lot to do with the families like I see them initially when the patient wants to start the process. I’ll speak to the patient, but I also speak to the family.* [#2] [Non-IO, Private] |
| 1. **Engaging in end-of-life discussions (including VAD)** | | Using VAD as an opportunity to engage in broader end-of-life discussions | *If a person asks about VAD, [the staff would] join in a discussion with them because it’s a perfect entry to your end-of-life care planning discussion. It saves [the practitioner from] bringing it up. “Now, it’s time for us to talk about difficult things, Mr. White. Have you thought about what’s going to happen, blah, blah?” So they bring it up. We’re into our discussion about their hopes and fears and what they might do and whatnot* [#3] [IO, Private]    *[VAD] often comes about as part of a broader discussion around end of life, and people are now starting to raise that as part of those discussions in ways that they never did before.  So then part of it’s been empowering staff to do the work that they would normally have done and not to let the arrival of VAD pivot them away from the work that they should be doing around those end-of-life discussions.* [#15] [IO, Private] |
|  |  | Inform patient of limitation of involvement | *So [practitioners are] very happy to discuss [VAD and end-of-life] because that’s part of your caring for the person, and [they] tell [patients] that [they]’ll stand by them whatever they choose to do. But [they] won’t be doing the Part 2 form [eligibility assessment] for them*. [#3] [IO, Private] |
|  |  | Escalate response to other colleagues | *A staff member can report up to their nursing manager or their manager and say, “Look, we’ve got somebody who’s raised VAD, or they’ve expressed a strong desire to die,”… So anyone that raises a desire to die gets referred to our service*. [#8] [IO, Public] |
| 1. **Eligibility assessments** | | Formal eligibility assessments | *We aren’t [an institution] that either assesses for or purposely facilitates VAD.* [#8] [IO, Public]    *We wouldn’t obstruct clinical staff coming in to do assessments … they’re free to do that.* [#16] [IO, Private] |
|  |  | Prognostication information | *[Staff] won’t [do the] Part 2 form [eligibility form] for them, but [they will] certainly provide [patients] with a prognosis letter*. [#3] [IO, Private] |
| 1. **Administration** | 1. **Self-administration** | Encouraging and facilitating community self-administration | *[Institution X] has assisted patients who have been discharged to the community because not every person, of course, needs to be in [the] hospital to undertake this.* [#6] [Non-IO, Private] |
|  |  | Medication management | *If the lethal substance is brought to the facility, none of our staff will take that into their personal possession. It belongs to the resident.* [#16] [IO, Private]  *I’m able to make up the medication for the patient if that’s what they’d like*. [#2] [Non-IO, Private]  *People can bring their substance into the hospital. We’ve got special drawers, bedside tables where people can put the medicine and access it themselves with a key we give them.* [#8] [IO, Public]  *Policy decisions had to be made about what to do with medications on-site… so I know that at other hospitals where, for instance, maybe patient autonomy was really prioritized above many other things … then perhaps the meds would be allowed to be right next to the patient at all times.* [#7] [Non-IO, Public] |
|  |  | Self-administration on premises | *We don’t impede somebody right at the end when they’ve actually decided – people can bring their substance into the hospital ... So we don’t feel that’s facilitating, we’re just not impeding what they decide … [We ask them if they can] at least just alert us to the fact, if and when they might be considering using it, so we can clinically, from a nursing point of view, make sure that things are prepared … We make sure they have certain medicines written on their drug chart if and when something goes wrong*. [#8] [IO, Public]    *So, any oral or any self-administered or physician-administered administration, [the VAD Coordinator or delegate is] present for*. [#2][Non-IO, Private]    *Consent forms [need] to be signed by the person to say they’re not going to take it without letting us know, because we want safety for our staff*. [#11][Non-IO, Private] |
|  | 1. **Practitioner administration** |  | *We wouldn’t allow somebody to come in and give the intravenous injection because that means that it has to be a registered medical staff member*. [#8][IO, Public]    *You can’t just join [Institution X] as a specialist and then go, “I actually came in to do voluntary assisted dying.”… the exception to that is if it’s a patient’s GP … [Institutional X will] provide [a] provisional appointment to a GP to come in and be part of that patient’s care delivery.* [#6] [Non-IO, Private] |
|  | 1. **Presence during administration** |  | *If the resident wanted us to be present at the time of ingesting the substance, we said to the staff, “You’ve got to just think about this very carefully. We do not expect you to be there.” So, from a company position, we are not expecting that.* [#16][IO, Private]    *We’ve even done that where we’ve called in their treating doctor to come and visit, a private visit, not as a clinician, to see the patient before they die or something like that, you know, just because it often, in the private system particularly, it’s often that relationship that’s been the important link.* [#8] [IO, Public]    *We always talked about, let’s have a case-by-case discussion if we ever receive these invitations to understand what’s the extent of our involvement … What would be our role there on the day, as in, what would the family’s expectations or client expectations be?* [#5] [Non-IO, Private] |
| 1. **Witnessing and verifying deaths** | | | *I’ve witnessed two [practitioner administrations]* [#11] [Non-IO, Private]  *If they can’t organize their own witnesses, we’ll organize [advocacy organization] to go as witnesses*. [#2] [Non-IO, Private]  *Our staff will, our nursing staff or our medical staff member, whoever’s most available, are able to go in and complete the verification of death after someone has enacted their Voluntary Assisted Dying Medication.* [#5] [Non-IO, Private] |
| 1. **Supports following a VAD death** | | Staff supports | *Throughout the process, we have staff support on the day. So we have a chaplain who is on-site throughout the whole day, even if it’s not their rostered day. They’ll be there, and they’re just usually in their office to talk to staff around that support and so forth. And then we also do a debrief as well after it, to see how people are going if there are any questions or any comments and so forth*. [#13] [IO, Private] |
|  |  | Support to patients’ families and loved ones | *I make sure that every family gets a phone call and we send them a card after [their loved one has undergone] voluntary assisted dying … and we offer them counselling with one of our … psychologists … and they’ve done more work in counselling patients’ families who have undergone voluntary assisted dying*. [#2][Non-IO, Private]  *We try to follow the family in as well…we do try to assist from our own resources. And then we also try to refer to community resources*. [#16] [IO, Private] |
|  |  | Supports to external staff | *In one of the situations, the doctor came out, and you can see that they weren’t their usual self.  And so, the chaplain said, “Can we help you in any way? Are you right?” So we talked to them, and we engaged with them to make sure that they were OK*. [#13] [IO, Private] |
